# Increase of Frequency and Modulation of Phenotype of Regulatory T Cells by Atorvastatin Is Associated with Decreased Lung Inflammatory Cell Infiltration in a Murine Model of Acute Allergic Asthma

**DOI:** 10.3389/fimmu.2016.00620

**Published:** 2016-12-21

**Authors:** Yurany Blanquiceth, Ana Lucia Rodríguez-Perea, Jorge H. Tabares Guevara, Luis Alfonso Correa, María Dulfary Sánchez, José Robinson Ramírez-Pineda, Paula Andrea Velilla

**Affiliations:** ^1^Grupo Inmunovirología, Departamento de Microbiología y Parasitología, Facultad de Medicina, Universidad de Antioquia UdeA, Medellín, Colombia; ^2^Grupo Inmunomodulación, Facultad de Medicina, Universidad de Antioquia UdeA, Medellín, Colombia; ^3^Sección de Dermatología, Departamento de Medicina, Facultad de Medicina, Universidad de Antioquia UdeA, Medellín, Colombia; ^4^Laboratorio de Patología, Laboratorio Clínico VID, Obra de la Congregación Mariana, Medellín, Colombia; ^5^Stanley S. Scott Cancer Center & Louisiana Cancer Research Center, Health Sciences Center, Louisiana State University, New Orleans, LA, USA

**Keywords:** regulatory T cells, IL-10, asthma, ovalbumin, atorvastatin

## Abstract

Regulatory T cells (Tregs) play an important role by controlling allergic inflammation of airways. Recently, it has been shown that statins have immunomodulatory properties, probably mediated by their effects on Tregs. Therefore, we evaluated the *in vivo* effect of atorvastatin (ATV) on Tregs and its association with the inflammatory process in a model of allergic asthma. BALB/c mice were sensitized with ovalbumin (OVA) and then challenged with intranasal OVA. ATV (40 mg/kg) was delivered by daily intraperitoneal injection for 7 or 15 days before each OVA challenge. ATV treatment for 7 days increased the frequency of Tregs in mediastinal lymph nodes (MLN) and the interleukin (IL)-10 in lungs. After 15 days of treatment, ATV increased the percentage of glucocorticoid-induced tumor necrosis factor receptor-related protein (GITR+) and programmed cell death protein 1 (PD-1+) Tregs in the lung, without enhancing their suppressive activity, but also increased the percentage of conventional T cells expressing GITR+, PD1+, and OX-40 (tumor necrosis factor receptor superfamily member 4). Although no significant changes were observed in the number of inflammatory cells in the bronchoalveolar lavage (BAL), OVA-specific immunoglobulin E in the serum, and type 2 helper (Th2) cytokines in the lungs, there was a significant decrease of peribronchial inflammation that negatively correlated with the Tregs in MLN and the concentration of IL-10 in the lung. These results suggest that ATV has an immunomodulatory role possibly mediated by their effects on Tregs, which could contribute to the control of inflammation during allergic asthma. Further studies are necessary to elucidate the contribution of Treg to immunomodulatory action of statins in the context of allergic asthma.

## Introduction

Allergic asthma is a highly complex chronic inflammatory disease of the airways. Since it is estimated that around 235 million people suffer from asthma, one of the objectives of the World Health Organization is to identify cost-effective interventions to reduce the morbidity and mortality related to this disease (1). Although immunopathology of asthma is heterogeneous, one of the first events that defines the initiation and perpetuation of chronic inflammation during allergic asthma is the differentiation of CD4^+^ T cells toward a type 2 helper cells (Th2) profile. Remarkably, these cells produce cytokines such as interleukin (IL)-5, which mediates the development and recruitment of eosinophils to the airways (2), and IL-4 and IL-13, which promote isotype switching to immunoglobulin E (IgE) (3). In addition, cells of the innate immunity, such as mast cells, type 2 innate lymphoid cells, and basophils, and cells of adaptive immunity, such as B cells, Th17 cells, and Th1 T cells, act together with epithelial cells to cause airway remodeling, leading to bronchial hyperresponsiveness, airway narrowing, edema and mucous hypersecretion, and structural changes characteristic of allergic asthma (4, 5). On the contrary, regulatory T cells (Tregs) constitute a subpopulation of CD4^+^ T cells that has been involved in controlling the magnitude of the immune response against environmental antigens during allergic asthma (6). Tregs express the transcription factor forkhead box P3 (FOXP3), which is critical for their development and function (7). During allergic asthma, Tregs seem to play a beneficial role by inhibiting Th2 responses and regulating B-cell antibody production, mainly through the secretion of IL-10 and transforming growth factor beta (TGF-β) (8). Likewise, Tregs through contact-dependent mechanisms inhibit mast cell degranulation through OX40-OX40L (tumor necrosis factor receptor superfamily member 4) (9). However, the role of other surface molecules mediating suppressive function in allergic asthma is more controversial, since it has been demonstrated that programmed cell death protein 1 (PD-1) and glucocorticoid-induced tumor necrosis factor receptor-related protein (GITR) could have a proasthmatic role (10, 11).

Currently, the main treatment for allergic asthma is glucocorticosteroids, which inhibit the expression of multiple inflammatory genes; however, their prolonged use may have adverse side effects (12) and around 5 to 10% of patients develop resistance to glucocorticoids (13). The search and development of alternative or complementary therapies, with a good safety profile, easy administration, and immunomodulatory potential, would constitute an attractive strategy in the control of immunopathology of asthma. Statins are drugs widely used to lower lipid levels, but they also possess cholesterol-independent or “pleiotropic” effects, among which are their potent immunomodulatory and anti-inflammatory action (14). Regarding immunomodulatory actions, recently, we and others have demonstrated that statins can have immunomodulatory actions mediated by their direct effect on Tregs, increasing the size of this population, their phenotype or function in the steady state (15), and under inflammatory conditions (16–18). Moreover, although statins possess immunomodulatory effects in murine models of ovalbumin (OVA)-induced asthma, such as the reduction of leukocytes in bronchoalveolar lavage (BAL) (19–21), OVA-specific IgE level (20), Th2-cytokine secretion (19–21), bronchial (peribronchial and perivascular) inflammation (19), and mucus production (21), it is unknown whether these beneficial effects are associated with the induction and/or activation of Tregs. Therefore, we hypothesized that pharmacologic treatment with atorvastatin (ATV) could contribute to reduce the inflammatory response of allergic asthma in a murine model by modulating the size, phenotype, or function of the Treg population. We found that ATV treatment increased Treg population but decreased the transcriptional expression of Th2 cytokines profile in mediastinal lymph nodes (MLN) and increased IL-10 levels in the lung, suggesting an immunomodulatory role of ATV, possibly mediated by Tregs, in controlling the peribronchial inflammation during allergic asthma.

## Materials and Methods

### Animals

The 8- to 10-week-old pathogen-free male BALB/c mice weighing 20–25 g were provided by the Sede de Investigación Universitaria vivarium of the University of Antioquia. They were maintained in laminar flow cage racks with a 12-hour light/dark cycle and *ad libitum* access to food and water. All experimental procedures were approved by the Ethics Committee for Animal Experimentation of the University of Antioquia.

### Experimental Design

#### OVA Sensitization

Male mice were divided into three groups with around five mice per group in each of three replicates. The sensitized (S) and ATV-treated (S + ATV) groups were obtained by using mice sensitizing with 2 µg OVA (Sigma-Aldrich, St. Louis, MO, USA) emulsified in 200 µL containing 2.6 mg/mL aluminum hydroxide (Sigma-Aldrich) by intraperitoneal (i.p.) injection on days 0 and 14 (22). The animals were subjected to two different protocols of OVA challenge and ATV treatment.

##### 27 Days Model

On days 20 and 24, the mice were challenged intranasally (i.n.) with 10 µg OVA dissolved in 50 µL phosphate-buffered saline (PBS) (23). The S + ATV group received an i.p. injection of 40 mg/kg ATV (Biogen Laboratory, Bogotá, Colombia) dissolved in PBS, which was administered 30 min before the OVA challenge from day 20 to 26. On day 27, the mice were sacrificed.

##### 35 Days Model

We performed four OVA challenges on days 20, 24, 28, and 32. Treatment with ATV was also performed daily, from day 20 to 34, for a total of 15 doses. On day 35, the mice were sacrificed (Figures S1A,B in Supplementary Material).

In addition, we included mice that were not sensitized (NS) but received the challenges with OVA according to each model, as control groups. In some experiments, we included ATV-treated NS mice.

### Determination of OVA-Specific Serum IgE Levels

Blood samples were collected immediately after anesthesia by cardiac puncture. Serum samples were obtained for the quantification of OVA-specific IgE antibodies by enzyme-linked immunosorbent assay (ELISA). Briefly, 96-well plates were coated with anti-mouse IgE antibodies overnight at 4°C (BD PharMingen, San Diego, CA, USA). ELISA plates were blocked for 2 h with 10% fetal bovine serum (FBS; Gibco-Thermo Fisher Scientific, USA) and 0.05% Tween 20 (AMRESCO, Solon, OH, USA) dissolved in PBS at room temperature. Then, 50 µL of serum was serially diluted fivefold in PBS, added to the wells, and incubated overnight at 4°C. Biotinylated OVA was added the following day, and the streptavidin/alkaline phosphatase complex (DAKO, Glostrup, Denmark) and the p-nitrophenylphosphate substrate (Sigma-Aldrich) were added and incubated for 20 min. To detect the reaction, the optical density was measured using an ELISA detector at 405 nm. Serum titers are expressed as the reciprocal value of the maximal serum dilution with a positive result defined as the optical density value higher than twofold over the background value of a serum sample at1:5 dilution from a non-sensitized mouse (22).

### Collection and Analysis of Bronchoalveolar Lavage

Immediately after blood collection, the thoracic cavity was opened by careful dissection. The trachea was exposed, and a small transverse incision was made just below the larynx, where a cannula was inserted. Then, BAL was collected by lavaging the lungs three times with 1 mL PBS. BAL was resuspended, and the total viable cell numbers were determined by trypan blue exclusion using a hemocytometer. For the preparation of cytospins, BAL was centrifuged at 600 × *g* for 10 min using a Cytospin III (Shandon, Pittsburg, PA, USA). These preparations were stained with colorant Wright, and differential cell counting of 100 cells was performed using standard morphological criteria.

### Cytokine Levels in Lung Tissues

Left lungs were macerated and filtered through a nylon mesh (70 µm; Spectrum Labs, Rancho Dominguez, CA, USA) in 2 mL PBS and centrifuged at 1,500 rpm at 4°C for 10 min. Supernatants were collected, and the IL-4, IL-5, and IL-10 levels were evaluated using the kit Mouse OptEIA (BD PharMingen) and IL-13 levels using the Mouse DuoSet ELISA (R&D Systems Inc., McKinley, MN, USA) according to the manufacturer’s instructions. The concentration of the cytokines was determined through standard curves generated using different concentrations of recombinant cytokines. The limit of detection for each cytokine was as follows: IL-4: 7.8 pg/mL, IL-5: 15.6 pg/mL, IL-13: 62.5 pg/mL, and IL-10: 31.3 pg/mL.

### Lung Histology

After BAL sampling was completed, the right lungs were removed from the thoracic cavity by careful dissection and fixed in buffered 10% formalin. After fixation, the lungs were sectioned and embedded in paraffin, and 4-µm sections were cut using a Leica microtome (Leica Microsystems, Nussloch, Germany). The sections were assigned a random code to blind the examiner identifying each specimen. Tissue sections were then stained with hematoxylin and eosin (H&E; Sigma-Aldrich). The degree of inflammation was quantified following a scale modified from that of Cao et al. (24): 0, normal; 1, few cells; 2, a ring of inflammatory cells 1 cell layer deep; 3, a ring of inflammatory cells 2–4 cells deep; 4, a ring of inflammatory cells 5–6 cells deep; and 5, a ring of inflammatory cells of >6 cells deep. To evaluate mucus production, tissue sections were stained with Periodic acid–Schiff (PAS). The number of goblet cells was determined using the formula: (PAS-positive cells/total cells) × 100. Mucus production was determined according to the following scale: 0: 0%; 1: ≤15%; 2: 16–30%; 3: 31–45%; 4: 46–60%; and 5: >60% goblet cells (24). The scores of the pulmonary inflammation and mucus production were performed on a microscope (Nikon Labophot 2; Melville, NY, USA) at a magnification of ×400 by examining at least eight representative fields as analyzed by a blinded pathologist.

### Collection of Lung and MLN Cells and Flow Cytometry

Mediastinal lymph nodes were removed and processed similarly to lung as described in Section “Cytokine Levels in Lung Tissues.” The cells were collected, and the remaining erythrocytes of the left lung were lysed (lysis buffer, eBioscience, San Diego, CA, USA) according to the manufacturer’s instructions. Single-cell suspensions from MLN and lung tissues were resuspended at 1 × 10^6^ cells/mL. The cells were labeled with the following monoclonal fluorochrome-conjugated antibodies: anti-CD3-Brilliant Violet 510 (clone 17 A2, BioLegend, San Diego, CA, USA), anti-CD4-eFluor 450 (clone RM4-5, eBioscience), anti-CD25-APC (clone PC61, BD Pharmigen), anti-OX-40-PE (clone OX-86, eBioscience), anti-PD-1-APC-eFluor 780 (clone J43, eBioscience), and anti-GITR-PE-Cyanine 7 (clone DTLA-1, eBioscience) for 30 min at 4°C in the dark. The cells were then washed in PBS and incubated with a fixation buffer (eBioscience) for 30 min at 4°C in the dark. After washing, the cells were resuspended in permeabilization solution 1× (eBioscience) and stained with anti-FOXP3-Alexa Fluor 488 (clone FJK-16s, eBioscience) for 30 min at 4°C in the dark. Flow cytometry was performed on a FacsCANTO II TM (BD, San Diego, CA, USA). At least 100,000 events were analyzed with FlowJo (Tree Star) or FACSDiva (BD Biosciences) software. Gating strategy is indicated in Figure S2 in Supplementary Material.

### Quantitation of Gene Expression Using Real-time Polymerase Chain Reaction (PCR)

Total RNA was purified from cell suspensions of MLN using a commercial kit (RNeasy mini kit, Qiagen, Hilden, Germany) according to the manufacturer’s instructions. The RNA was retrotranscribed to cDNA using random hexamers and the Revertaid™ H Minus First Strand cDNA Synthesis Kit (Fermentas, Glen Burnie, MD, USA). PCRs were performed in a final volume of 15 µL using the Maxima SYBR Green qPCR master mix kit (Fermentas). Specific primers and the PCR conditions are shown in Table S1 in Supplementary Material. Real-time reverse transcription PCR was performed in a CFX96™ Real-Time PCR Detection System (Bio-Rad, Hercules, CA, USA). The data are expressed as relative units of each gene normalized against the constitutive gene (β-actin) using the formula 1.8^−[∆Ct]^, where 1.8 corresponds to the mean PCR efficiency of 80% (25).

### *In Vitro* Suppression Assay

CD4^+^ T cells were obtained from the splenocytes and purified using the CD4^+^CD25^+^ regulatory T Cell Isolation Kit (Miltenyi Biotec, Auburn, CA, USA), following the manufacturer’s instructions. CD4^+^CD25^+^ Tregs were cultured in 96-well round-bottom plates and preactivated with plate-bound anti-CD3 (2.5 µg/ml, 145-2C11, eBioscience) and soluble anti-CD28 (2.5 µg/mL, 37.51, eBioscience) antibodies in RPMI 1640 medium (Sigma-Aldrich) supplemented with 10% FBS, 100 U/mL IL-2 obtained from the Institutes of Health, AIDS Research and Reference Reagent Program (Bethesda, MD, USA), 100 U/ml penicillin, and 100 µg/mL streptomycin (Sigma-Aldrich) for 48 h at 37°C with 5% CO_2_. The Tregs were then co-cultured with CD4^+^CD25^−^ conventional T cells (Tcon) labeled with 1.25 µM carboxyfluorescein diacetatesuccinimidyl ester (CFSE, Invitrogen) and stimulated as indicated earlier at a ratio of 1:1 in 96-well plates. Co-cultures were incubated for 72 h at 37°C with 5% CO_2_. Cell division was measured by CFSE dilution through flow cytometry. The percentage of suppression was calculated as follows: (100 − (% proliferation of Tcon in the presence of Treg/% proliferation of Tcon alone) × 100).

### Statistical Analysis

The data were analyzed by Student’s *t*-test and are expressed as the mean ± SD. When the data were not normally distributed, the Mann–Whitney test was applied and the results are expressed as the median ± interquartile range. The program GraphPad Prism Version 6 was used for the analysis (San Diego, CA, USA). Correlation analyzes were performed using the Spearman coefficient. A value of *p* < 0.05 was considered statistically significant.

## Results

### ATV Increases the Frequency of Tregs in MLNs

**Figure 1:**
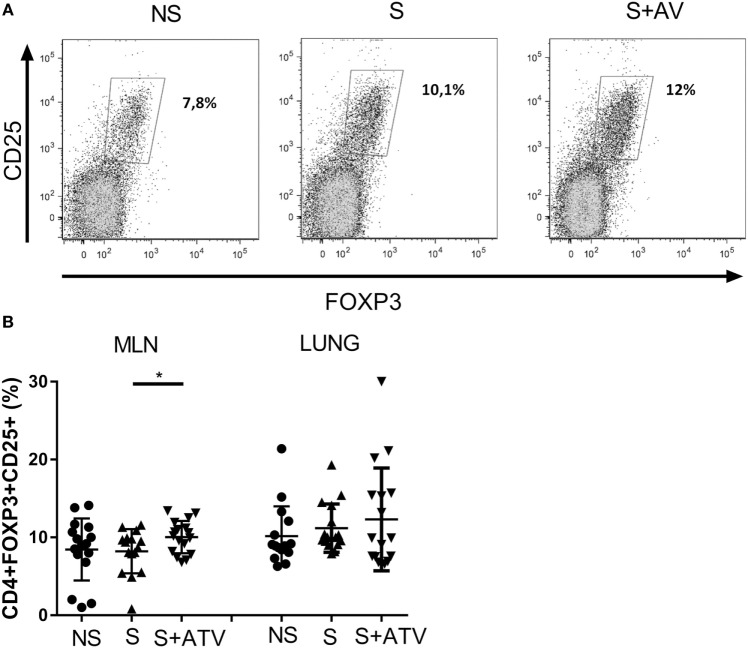
**Atorvastatin (ATV) increases the frequency of CD4^+^FOXP3^+^CD25^+^ regulatory T cells (Tregs) in a murine model of allergic asthma**. **(A)** Representative flow cytometry dot plot of CD25^+^FOXP3^+^ Tregs gated on CD4^+^ of mediastinal lymph nodes (MLN) from non-sensitized (NS●), sensitized (S▲), and sensitized treated with ATV (S + ATV▾) mice evaluated at day 27. **(B)** Frequency of CD4^+^CD25^+^FOXP3^+^ Treg in MLN and lung in the study groups. Data are shown as mean ± SD (*n* = 14–17 mice per group pooled from three independent experiments, *p < 0.05 Student’s *t*-test).

Since a beneficial role of Tregs has been demonstrated during asthma (26–28), and because ATV treatment increases Treg populations, we evaluated the immunomodulatory effect of ATV treatment on Tregs in a murine model of allergic inflammation of the airways. As shown in Figure 1A, treatment with ATV (40 mg/kg, i.p.) for 7 days significantly increased the frequency of CD25^+^FOXP3^+^ Tregs in MLN. No significant difference was observed between groups in the lung tissue (Figure 1B). Interestingly, ATV treatment also increased the frequency of Treg in MLN and lung in non-sensitized mice compared to untreated non-sensitized animals (Figure S3 in Supplementary Material).

### Long-Lasting Treatment with ATV Modulates the Phenotype of the Treg Population

**Figure 2:**
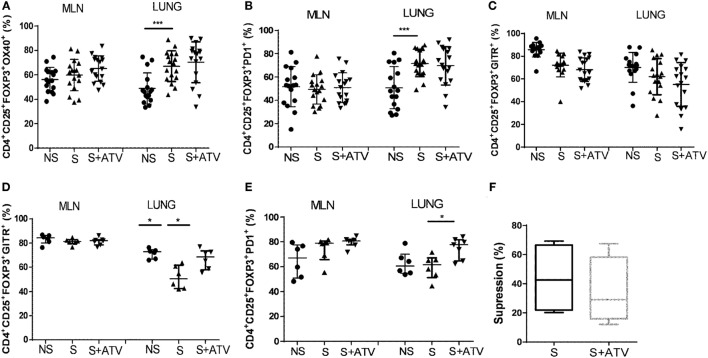
**Long-lasting atorvastatin (ATV) treatment modulates phenotype of regulatory T cells (Tregs) in a murine model of asthma**. The frequency of **(A)** CD4^+^CD25^+^FOXP3^+^OX40^+^ Tregs, **(B)** CD4^+^CD25^+^FOXP3^+^PD1^+^ Tregs, and **(C)** CD4^+^CD25^+^FOXP3^+^GITR^+^ Tregs was measured by flow cytometry in mediastinal lymph nodes and lung on day 27 in non-sensitized (NS●), sensitized (S▲), and sensitized treated with ATV (S + ATV▾) mice. The frequency of **(D)** CD4^+^CD25^+^FOXP3^+^GITR^+^ Tregs and **(E)** CD4^+^CD25^+^FOXP3^+^PD1^+^ Tregs was measured by flow cytometry in both tissues in the study groups at day 35. **(F)** Percentage of suppression of conventional T cells proliferation in the presence of Tregs from ATV-treated or untreated sensitized mice (*n* = 4 mice per group). Data from **(A–C)** are shown as mean ± SD (*n* = 12–17 mice per group pooled from three independent experiments, *p < 0.05 Student’s *t*-test). Data from **(D–F)** are shown as median and interquartile range (*n* = 6–7 mice per group from one experiment, *p < 0.05, ***p < 0.001 Mann–Whitney test).

Since differential expression of surface molecules on Tregs can be associated with their regulatory function, we examined whether ATV could modulate the phenotype of Tregs during allergic inflammation of the airways. It was observed that sensitization *per se* increased the frequency of Tregs expressing OX-40 and PD-1 molecules in lung compared to non-sensitized mice (Figures 2A,B). Although ATV treatment for 7 days did not affect the expression of OX-40, PD-1, or GITR on Tregs in both MLN and lung of sensitized mice compared to the untreated sensitized mice (Figures 2A–C), ATV upregulated the percentage of Treg expressing OX-40 and PD-1 in lung of non-sensitized mice (Figure S4 in Supplementary Material).

Considering that changes in the phenotype of Tregs could depend on time (15), we increased the number of doses of ATV from 7 to 15 and observed a significantly increased frequency of Tregs expressing GITR and PD-1 in the target tissue (lung) compared to untreated sensitized mice (Figures 2D,E). Also, upregulation of the OX-40, PD-1, and GITR molecules was observed on conventional CD4^+^ T cells (FOXP3- population) after ATV treatment (Figure S5 in Supplementary Material). In spite of increasing the expression of suppression markers on Tregs, the suppressive activity after 15 days of ATV treatment was not enhanced, as shown in the classical *in vitro* suppression assay (Figure 2F; Figure S6 in Supplementary Material).

### ATV Increases *Foxp3* and *TGF-*β Messenger RNA in MLN and IL-10 Levels in the Lung

**Figure 3:**
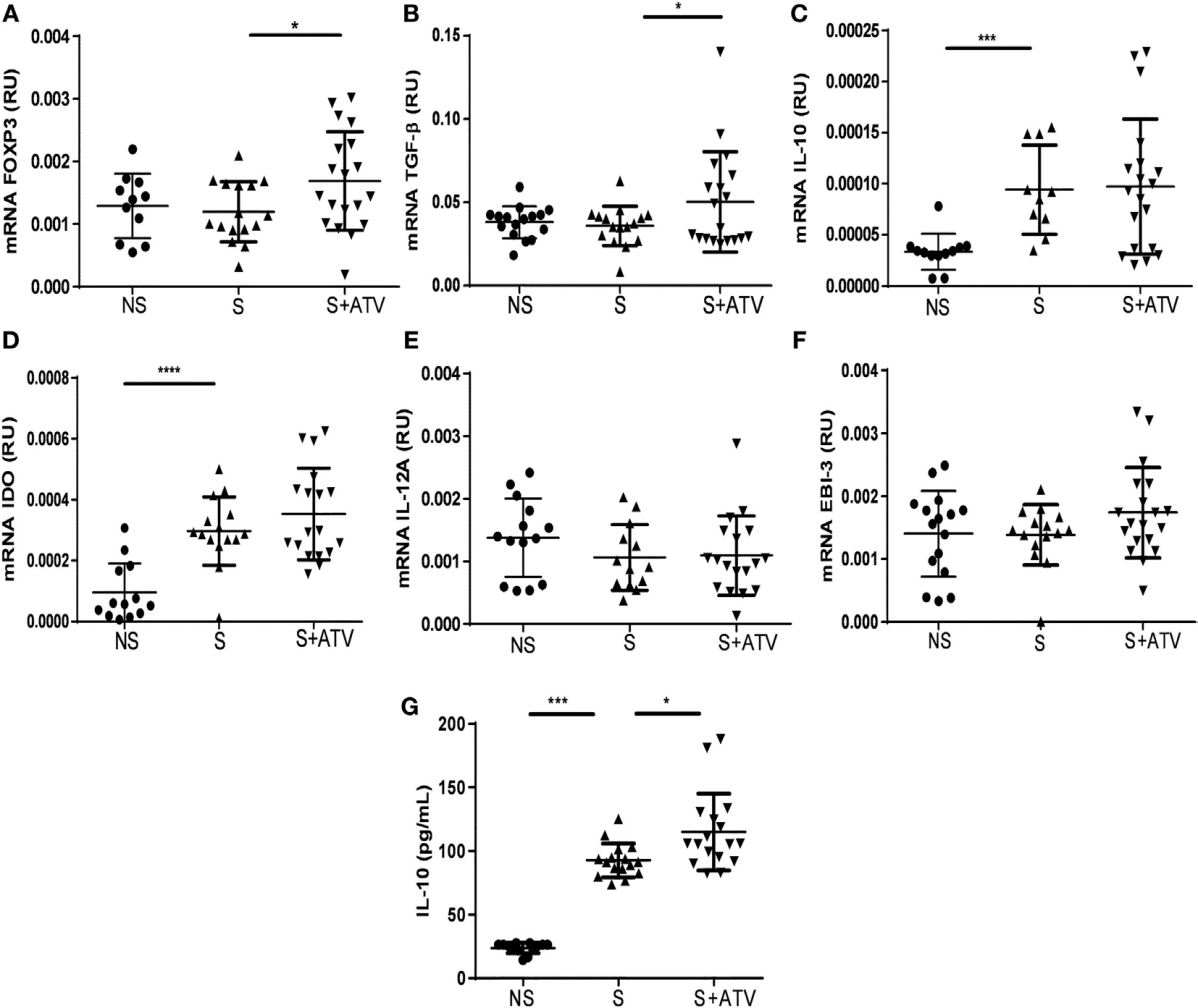
**ATV increases forkhead box P3 (*Foxp3*) and transforming growth factor beta (*TGF-*β) mRNA in MLN and the levels of interleukin-10 (IL-10) in lung in a murine model of asthma**. mRNA expression of **(A)** *Foxp3*, **(B)** *TGF-*β, **(C)** *IL-10*, **(D)** *IDO*, **(E)** *IL-12A*, and **(F)** *EBI-3* relative to the β-actin housekeeping gene was obtained from cell suspension of MLN recovered from non-sensitized (NS●), sensitized (S▲), and sensitized treated with ATV (S + ATV▾) mice sacrificed on day 27. **(G)** Supernatants from cell suspension of lung were obtained from study groups, and the levels of IL-10 were evaluated by ELISA. The data are shown as mean ± SD (*n* = 10–19 mice per group pooled from three independent experiments, *p < 0.05, ***p < 0.001, ****p < 0.0001 Student’s *t*-test).

To further confirm the *in vivo* effect of ATV on the Tregs under allergic inflammatory conditions, we evaluated the transcriptional expression of some genes associated with Treg function in MLN. Administration of ATV led to a significant increase in *Foxp3* and *TGF-*β mRNA expression in sensitized animals compared to sensitized but untreated mice (Figures 3A,B). Sensitization *per se* induced a significant increase in *IL-10* and indoleamine-pyrrole-2, 3-dioxygenase enzyme (*IDO)* mRNA level (Figures 3C,D). However, ATV treatment had no effect on the transcriptional level of *IL-10, IDO*, or *IL-35*, the latter composed of two subunits: *IL-12A* and *EBI-3* (Figures 3C–F, respectively).

Because IL-10 is involved in controlling allergic asthma (8, 29) and its production could be promoted by statins (30, 31), we assessed the IL-10 concentration in lung homogenates. Although sensitization increased the level of IL-10 compared to that of non-sensitized mice (Figure 3G), ATV treatment further boosted IL-10 production (Figure 3G).

### ATV Therapy Modulates the Inflammatory Process in Sensitized Animals

**Figure 4:**
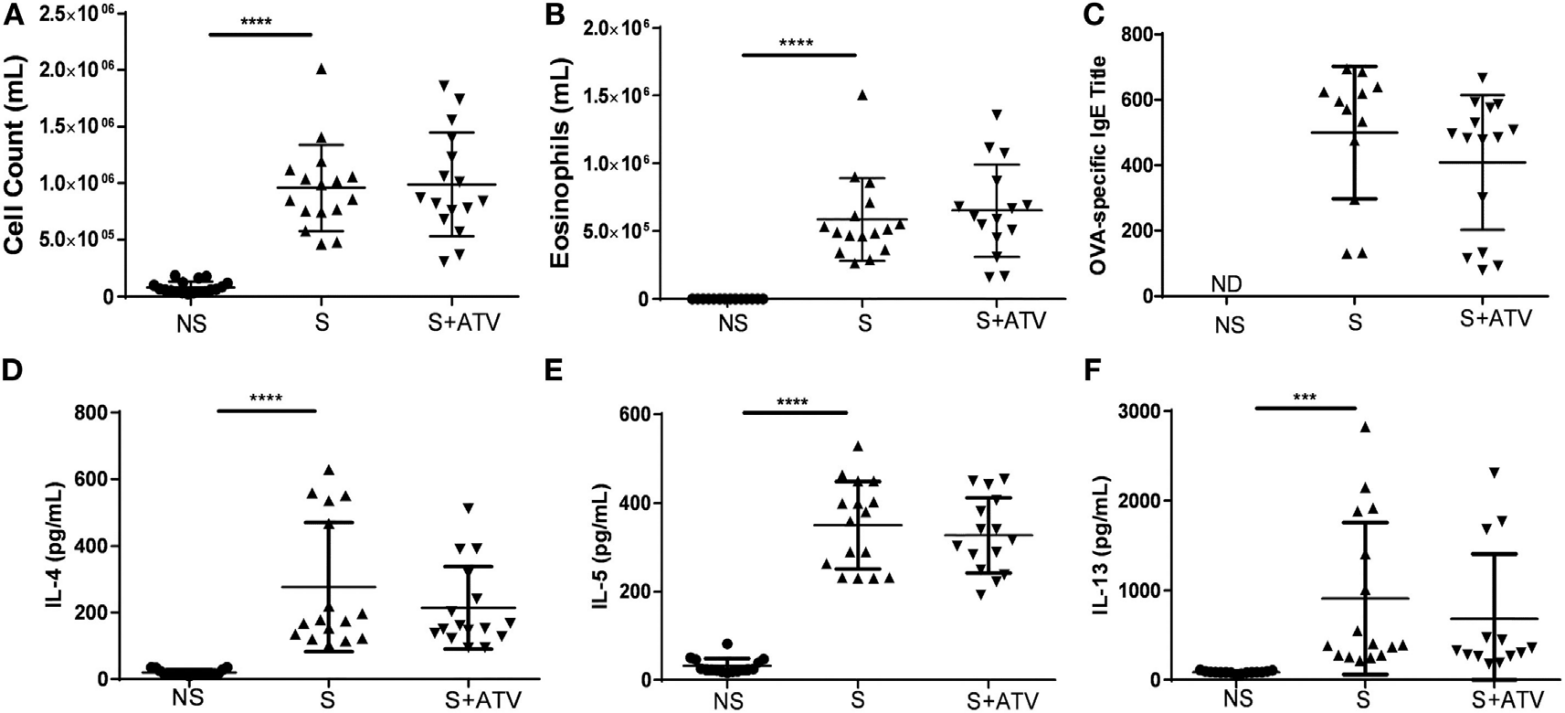
**Effect of atorvastatin (ATV) treatment in the type 2 helper cell (Th2) inflammatory response in a murine model of asthma**. **(A)** Bronchoalveolar lavage (BAL) cell count was determined using an hemocytometer by diluting the sample1:2 in trypan blue, whereas **(B)** eosinophils were determined after cytocentrifugation of BAL and staining with Wright by differential cell count using standard morphological criteria in non-sensitized (NS●), sensitized (S▲), and sensitized treated with ATV (S + ATV▾) mice sacrificed on day 27. **(C)** Ovalbumin (OVA)-specific immunoglobulin E in serum and **(D)** cytokines such as interleukin (IL)-4, **(E)** IL-5, and **(F)** IL-13 in lung homogenate were measured by ELISA in the study groups. Data are shown as mean ± SD (*n* = 13–17 mice per group pooled from three independent experiments, *p < 0.05, ***p < 0.001, ****p < 0.0001 Student’s *t*-test). ND: not detected.

Previous investigations have demonstrated the anti-inflammatory activity of statins during allergic asthma (19, 20, 32). Therefore, we examined the effects of ATV on the influx of inflammatory cells into BAL in OVA-sensitized mice. As expected, there was a significant increase in the number of inflammatory cells in sensitized versus non-sensitized mice (Figure 4A). Similarly, sensitization induced a significant increase in eosinophil count compared to non-sensitized animals (Figure 4B). However, in our model, ATV treatment did not reverse cell infiltration or eosinophil count induced by sensitization (Figures 4A,B). As expected, the sensitization process also increased the serum OVA-specific IgE levels and the IL-4, IL-5, and IL-13 concentrations in lung supernatants compared to control mice (Figures 4C–F, respectively). However, ATV treatment did not significantly modify the serum IgE levels or Th2 cytokines in the lung compared with sensitized mice (Figures 4C–F).

**Figure 5:**
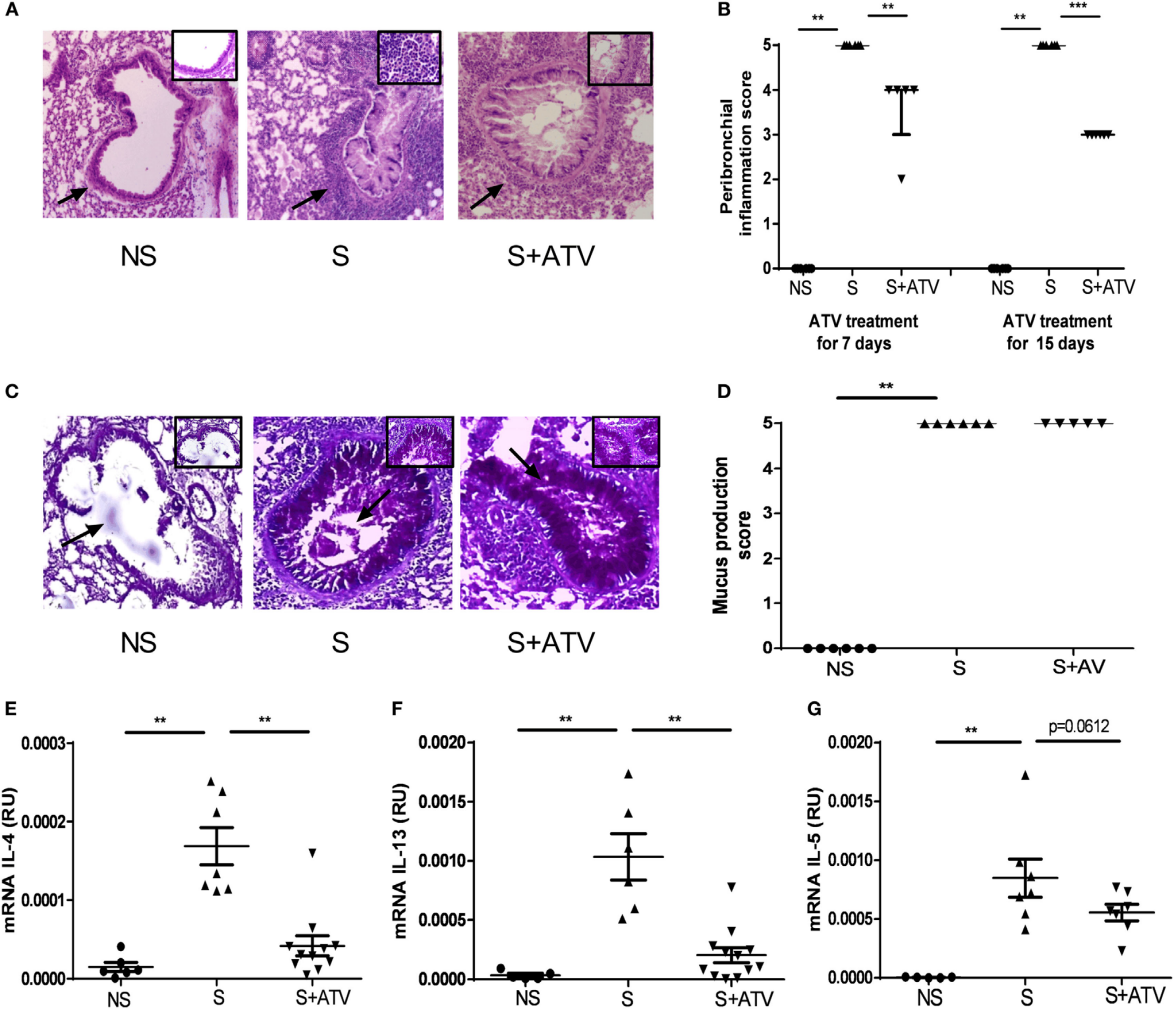
**Atorvastatin (ATV) modulates the inflammatory process *in situ* in a murine model of asthma**. **(A)** A representative hematoxylin and eosin section from non-sensitized (NS●), sensitized (S▲), and sensitized treated with ATV (S + ATV▾) mice showing peribronchial inflammatory infiltrates after 7 days of ATV treatment; magnification (×40) and box (×400), the arrows indicate the peribronchial region. **(B)** Score of peribronchial inflammation was measured on day 7 or 15 of ATV treatment using the scale described in Section “Materials and Methods.” The data are shown as median and interquartile range of *n* = 5–8 mice per group from one experiment. **p < 0.01, ***p < 0.001 (Mann–Whitney test). **(C)** A representative Periodic acid–Schiff staining section from each study group showing mucus production after 7 days of ATV treatment; magnification (×40) and box (×400), the arrows indicate the mucus in the lumen of the bronchus. **(D)** Score of mucus production was measured on day 7 of ATV treatment using the scale described in Section “Materials and Methods.” mRNA expression of **(E)** interleukin (IL)-4, **(F)** IL-13, and **(G)** IL-5, relative to the β-actin housekeeping gene was obtained from cell suspension of mediastinal lymph nodes in the study groups. Data from **(D,E)** are shown as mean ± SD (*n* = 5–12 mice per group pooled from three independent experiments, **p < 0.01 Student’s *t*-test).

We next examined the lung tissue sections stained with H&E and observed that OVA-sensitized mice displayed extensive inflammatory infiltrates in peribronchial areas compared to non-sensitized animals (Figures 5A,B). Interestingly, ATV treatment for 7 and 15 days reduced the score value of inflammation in the peribronchial region from 5 in ATV-untreated sensitized mice to 4 and 3 in those treated, respectively (Figure 5B). Although mucus production was observed in sensitized mice compared to non-sensitized mice, ATV treatment did not modulate this parameter (Figure 5C).

The mRNA expression level of Th2 cytokines in MLN was then compared: it showed that the sensitization process also increased mRNA expression of all evaluated cytokines compared to that in non-sensitized mice, whereas that ATV treatment significantly decreased the mRNA of IL-4 and IL-13 (Figures 5D,E). A trend toward decrease was also observed in the level of IL-5 after ATV treatment (Figure 5F).

### The Induction of Tregs Correlates with a Decrease of Peribronchial Inflammation

**Figure 6:**
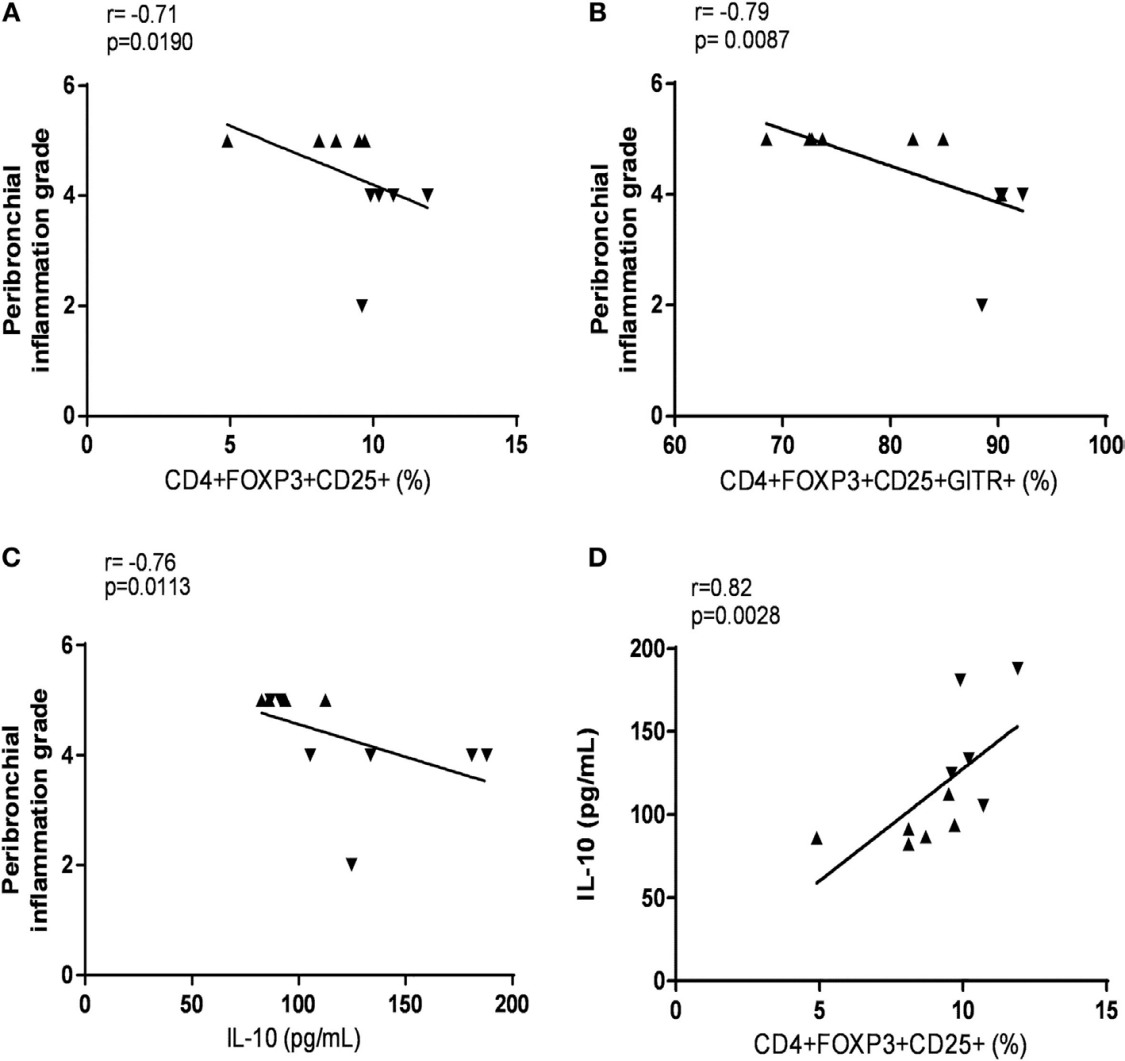
**The induction of regulatory mechanisms by atorvastatin (ATV) are correlated with the decrease of peribronchial inflammation in a murine model of asthma**. Peribronchial inflammation was negatively correlated with **(A)** CD4^+^CD25^+^FOXP3^+^ cell frequency in mediastinal lymph nodes (MLN), **(B)** CD4^+^ CD25^+^FOXP3^+^GITR^+^ cell frequency in MLN, and **(C)** interleukin (IL)-10 levels in the lung, whereas **(D)** IL-10 levels in the lung were positively correlated with CD4^+^CD25^+^FOXP3^+^ cell frequency in MLN. Correlation analysis was determined by Spearman’s correlation coefficient; *n* = 5–6 mice per group. Sensitized mice: ▲ and ATV-treated mice: ▾.

To determine whether Tregs contribute to the control of inflammation in lung tissue, we correlated the frequency of Tregs with several inflammatory parameters. Interestingly, a negative correlation between peribronchial inflammation and (i) frequency of Tregs in MLN (Figure 6A), (ii) frequency of Tregs expressing GITR in MLN (Figure 6B), and (iii) concentration of IL-10 in the lung (Figure 6C) was observed. Moreover, the IL-10 protein concentration in the lung was positively correlated with the frequency of Tregs in the MLN (Figure 6D).

## Discussion

Despite studies showing that statins have beneficial effects during asthma by decreasing inflammation in the lung, it is unknown whether these effects could be mediated by their action on Tregs. In this study, by using a murine model of allergic inflammation of the airways, we show that ATV treatment leads to a higher frequency and phenotypic changes in Tregs, along with high IL-10 levels, which could be limiting peribronchial inflammation.

Interestingly, ATV treatment for 7 days increased the CD25^+^FOXP3^+^ Treg population in lung-draining lymph nodes of sensitized animals (Figure 1B), suggesting that lymph node–resident conventional CD4^+^ T cells could be converted into Tregs, since conversion to Tregs might be mediated by statins as has been reported (17, 33). It is thought that statins are repressors of several signaling pathways that negatively regulate FOXP3 induction, either by blocking IL-6 signaling (34) or inhibiting the negative regulators of TGF-β signaling (33) and/or probably by downregulating the expression of methyltransferases (35), thus promoting the conversion of conventional CD4^+^ T cells into Tregs. Indeed, in models of autoimmunity (36) and delayed hypersensitivity (37), statins induce an increase in the Treg population, particularly in lymph nodes, which are niches favoring Treg induction (38).

In addition, the increase of *TGF-*β mRNA expression could be involved in statin-mediated changes of the size of the Treg population (39) since TGF-β promotes FOXP3 expression *via* Smad-dependent mechanisms (40). Alternatively, changes in the migration patterns of Tregs could explain this preferential enrichment in lymphoid tissues, because of the modulation of chemokines and adhesion molecules by statins (20, 37, 41). Remarkably, this Treg accumulation in lymphoid tissue could be critical for the establishment and regulation of an efficient antigen-specific immune response. In fact, by using a mouse model of chronic asthma, Carson et al. (26) showed that the resolution of inflammation is correlated with the accumulation of Treg cells in lung-draining hilar lymph nodes. On the basis of increase of Tregs in MLN, we found an increase of *Foxp3* mRNA expression in this compartment (Figure 3A), confirming the ATV effect on Tregs, as demonstrated in the previous studies of murine models of acute inflammation (37, 42). We characterized the Treg population by measuring the expression of several molecules, such as OX-40, GITR, and PD-1, whose suppressive capacities have also been implicated during allergic asthma. Interestingly, the treatment with ATV for 7 days did not change the Treg phenotype in MLN (Figures 2A–C); however, when it was administered for 15 days, there resulted a higher percentage of Tregs expressing GITR and PD-1 in the lung (Figures 2D,E), suggesting an activation state of these cells, particularly in this tissue. Nonetheless, we could not rule out that mononuclear cells trapped in blood vessels of lungs could have influenced these results since mice were not perfused with PBS. Previously, in both humans (43) and murine models of asthma (44), the importance of these molecules in attenuating inflammation was reported, since patients with severe asthma have a lower frequency of Tregs expressing GITR compared with healthy controls (43). We hypothesize that these phenotypic changes could be attributable to the longer duration of the treatment allowing the upregulation of other genes downstream of *Foxp3*, such as GITR (45). In agreement with these results, we previously found that shorter treatments with statins increased Treg frequency, whereas longer treatments induced phenotypic changes (15); this could turn on regulatory mechanisms to maintain a tight control on the size and functions of the Treg population (46) since an imbalance in this population could have detrimental effects on infections (47) and in tumor immunosurveillance (48). Supporting the effect of ATV on Tregs, we also found that naive non-sensitized mice treated exhibit higher frequency of Treg in MLN and lung, as well as Treg expressing OX-40 and PD-1 in lung, suggesting that ATV treatment also modulate their frequency and phenotype in non-inflammatory conditions (Figure S4 in Supplementary Material).

In addition, ATV did not modify the mRNA expression of *IDO* and *IL-35* in MLN (Figures 3D–F), which are molecules capable of attenuating the inflammatory process in allergic asthma models (49–51); since we only evaluated the transcriptional expression of such factors, we do not rule out the possibility that these cytokines could be increased at the protein level.

The effect of ATV on the suppressive function of Tregs appears more controversial (16, 17, 52, 53): we as well as others did not observe that statins increased the suppressive activity of Tregs (Figure 2F; Figure S6 in Supplementary Material). Thus, at least in the times we evaluated and in a non-antigen-specific manner, the phenotype observed did not confer a functional advantage for Tregs.

Interleukin-10 produced by Tregs constitutes one of the main suppression mechanisms during asthma (29). Interestingly, ATV treatment further significantly increased the levels of IL-10 induced by sensitization in the lung (Figure 3G) that was positively correlated with Tregs in MLN (Figure 6D), suggesting that in our model one of the main IL-10-producing cells could be Treg. However, we cannot exclude the contribution of other regulatory cells with the capacity to produce IL-10, such as Tr1 cells and M2 macrophages, considering that these cell populations can also be modulated by statins (30, 31). The importance of these findings is based on the fact that patients with asthma produce less IL-10, suggesting the fundamental role of the cytokine as mediator of immunological tolerance in the airways (54). Both ATV (55) and CD25^+^FOXP3^+^ Treg-producing IL-10 have been associated with decreases in the Th2-type allergen-specific immune responses in the airways (29). However, we observed no reduction of the Th2 response (Figure 4), probably because i.n. OVA administration leads to increased exposure and absorption of the antigen in the lungs, causing a higher intensity of inflammatory response compared to the aerosolized challenge (56). Likewise, regardless of ATV treatment, this high grade of inflammation could also explain why Tcon proliferation was not further suppressed by Tregs from ATV-treated mice, considering also that the upregulation of GITR and OX-40 on Tcon (Figure S5 in Supplementary Material) can confer to them resistance to suppression by Tregs (57, 58) and also the expression of these molecules maintains the Th2 inflammatory response (59, 60).

In contrast to the generalized inflammation observed in BAL, a more detailed analysis of lung tissue revealed that ATV treatment significantly reduces the accumulation of eosinophils and lymphocytes in peribronchial regions induced by sensitization (Figures 5A,B). Furthermore, a negative correlation between MLN-Treg and peribronchial inflammation was noted (Figure 6A), suggesting an important role of lymphoid nodules in controlling the effector phase of allergic asthma, as reported (26). Likewise, since ATV reduced the levels of IL-4 and IL-13 mRNA in lymph nodes (Figures 5E–G) and since IL-13 mRNA positively correlated with peribronchial inflammation (data not shown), Tregs possibly control the differentiation of effector T cells in these tissues rather than inhibiting T cell proliferation. Unfortunately, we cannot measure hyperresponsiveness, an important parameter in experimental asthma; however, it is important to note that ATV, through Treg-independent mechanisms, also decreases hyperresponsiveness and remodeling of the airways (55).

Previous researches have demonstrated that another statin like simvastatin and rosuvastatin reduce the majority of inflammatory markers (eosinophils in BALF, type 2 cytokines in lung, OVA-IgE in serum, and mucus production) and that ATV only modulates the inflammation when it is administrated from the beginning of sensitization phase until end of the OVA challenges (61). Therefore, the discrepancies between our results and these findings could be due to differences in pharmacokinetics among the statins used or to differences in the scheme of the treatment (number of doses, administration routes, concentration, and prophylactic or therapeutic administration of the drug) (19, 21, 55, 61). In summary, our data support an immunomodulatory role of statins mediated by their effect on Tregs in the context of allergic asthma. Treg accumulation in lung-draining lymph nodes could result from preferential presentation of OVA in lymphoid tissues rather than in non-lymphoid organs, such as the lung. In addition, Treg location in the target tissue enables them to secrete IL-10 and reduce peribronchial inflammation, and on the basis of the results obtained in this study, we propose a model shown in Figure S7 in Supplementary Material. These results suggest that ATV has an immunomodulatory role during allergic asthma possibly mediated by Tregs, since a negative correlation between the frequency of Tregs and peribronchial airway inflammation was found. However, further studies are necessary to deeply characterize the airway infiltrate and the molecular mechanisms involved in inflammation resolution after ATV treatment.

## Author Contributions

AR-P, PV, JR-P, and JT were responsible for conception and design of the study. YB and AR-P performed experiments, analyzed data, and wrote the manuscript. JT was in charge of the care, breeding, and maintenance of laboratory animals. LC performed histological analysis. PV, MS, and JR-P interpreted data and supervised the study. All the authors read and approved the final manuscript.

## Conflict of Interest Statement

The authors declare that the research was conducted in the absence of any commercial or financial relationships that could be construed as a potential conflict of interest.

## Abbreviations

Treg, regulatory T cells; ATV, atorvastatin; OVA, ovalbumin; MLN, mediastinal lymph nodes; IgE, immunoglobulin E; GITR, glucocorticoid-induced tumor-necrosis-factor-receptor-related protein; PD-1, programmed cell death protein 1; OX40, tumor necrosis factor receptor superfamily member 4; BAL, bronchoalveolar lavage; Th2, type 2 helper cells.
