# Supplementary material for: Increase of Frequency and Modulation of Phenotype of Regulatory T Cells by Atorvastatin Is Associated with Decreased Lung Inflammatory Cell Infiltration in a Murine Model of Acute Allergic Asthma
Source: Front Immunol. 2016 Dec 21;7:620. doi: 10.3389/fimmu.2016.00620 (PMC5174085; doi:10.3389/fimmu.2016.00620)
Supplement: Supplementary file 1 [file Data_Sheet_1.PDF]

Supporting Information

A. Model for 27 days

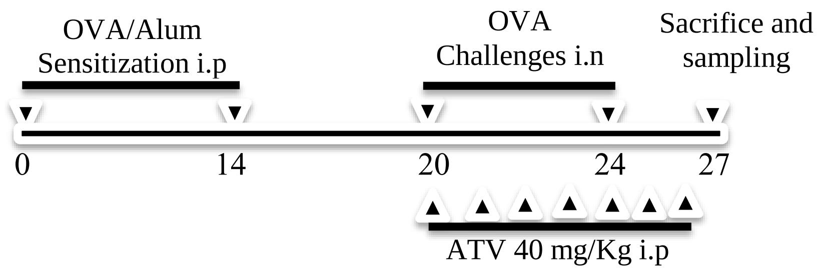

B. Model for 35 days

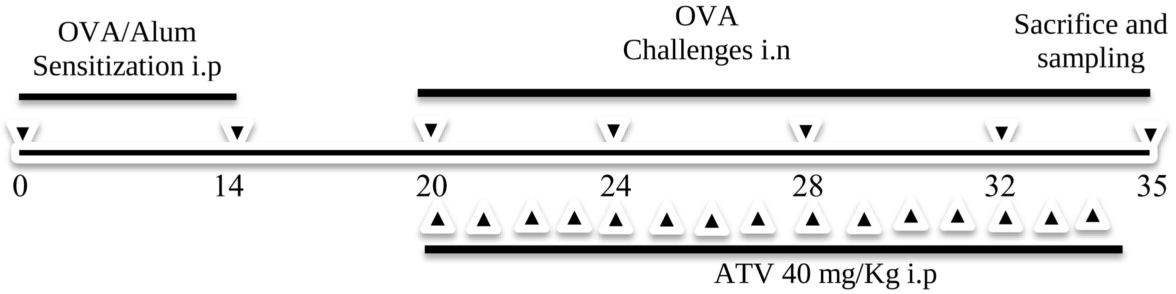

Supporting Information Figure 1. Experimental procedure. BALB/c mice were sensitized to OVA by i.p. injections of 2 µg OVA emulsified in 200 µL of PBS containing 2.6 mg of aluminum hydroxide ($Al(OH_3)$) adjuvant on days 0 and 14. Non-sensitized mice were used as control group. On day 20 and 24 all animals were challenged with 10 µg of OVA dissolved in PBS via i.n. **(A)** 40 mg/kg of ATV dissolved in PBS were administered via i.p., 30 min before the OVA-challenge from day 20 to day 26 in the 27-day model. **(B)** The same scheme of ATV as **(A)** from day 20 to day 34, in the 35-day model. All mice were sacrificed 24 h after the last dose of ATV.

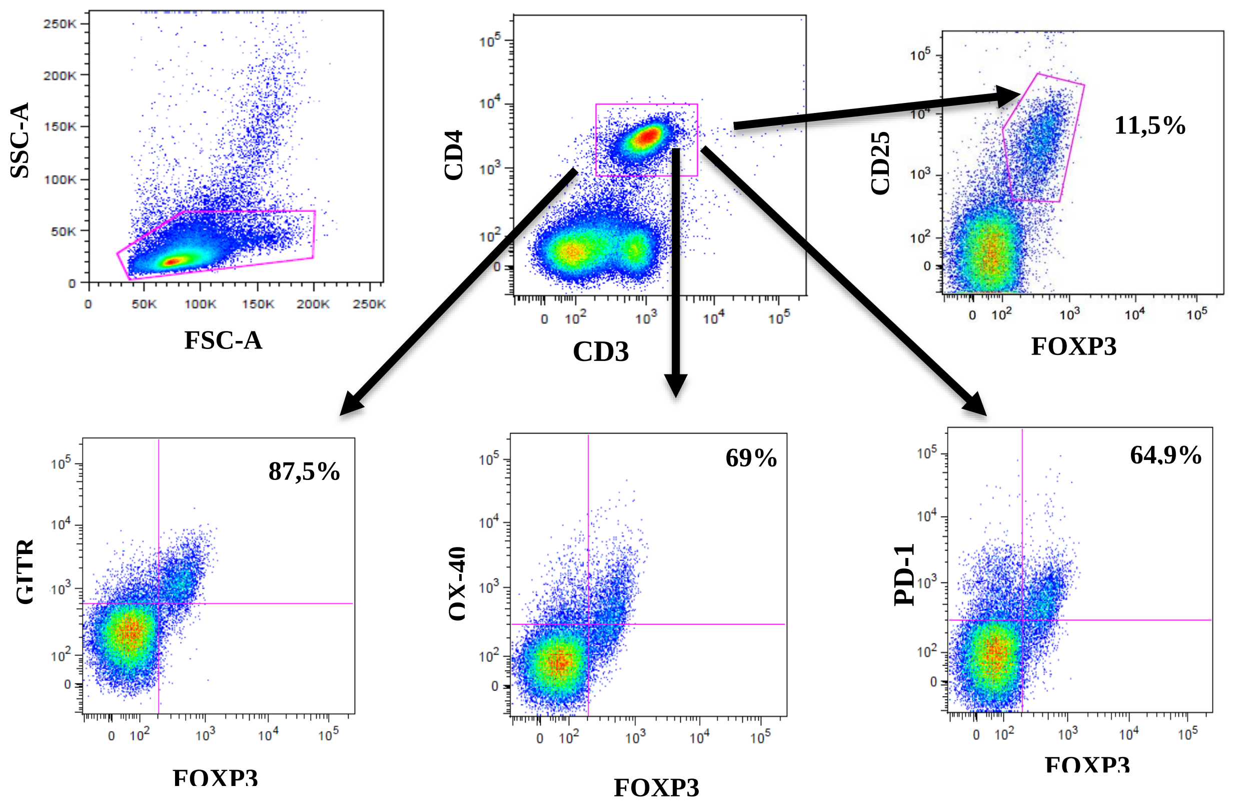

Supporting Information Figure 2. Gating strategy for selection of regulatory T cells. Representative analysis of the strategy used to identify Tregs in MLN and lung by multi-parametric flow cytometry. Lymphocytes were gated on characteristics of forward scatter (FSC-A) and side scatter (SSC-A), then double positive T cells for CD3 and CD4 were gated. Regulatory T cells were identified according to the expression of CD25 and FOXP3, and the percentage of expression of PD-1, OX40 and GITR was determined on Treg cells.

Supporting Information. Table 1. Specific primers and profiles for real-time PCR

**Gen**	**Primer**	**PCR thermal profile**	**Product Length (bp)**
IL-4	Fw 5` AGGAGAAGGGACGCCATGC 3`<br>Rv 5`CTCTGTGGTGTTCTTCGTTGC 3`	40 cycles 95ºC 0:08 sec, 58ºC 30 sec and 72ºC 1 min.	70
IL-5	Fw 5` AATGAGACGATGAGGCTTCCTG 3`<br>Rv 5` ACACTTCTCTTTTTGGCGGTCAAT 3`	40 cycles 95ºC 0:08 sec, 58ºC 30 sec and 72ºC 1 min.	177
IL-13	Fw 5´ACAGCTCCCTGGTTCTCTCA 3´<br>Rv 5´ ACCACCAAGGCAAGCAAGAG 3´	40 cycles 95ºC 0:08 sec, 58ºC 30 sec and 72ºC 1 min.	83
Foxp3	Fw` 5` CTC ATG ATA GTG CCT GTG TCC TCA A 3`<br>Rv`5` AGG GCC AGC ATA GGT GCA AG 3`	40 cycles 95ºC 0:08 sec, 58ºC 30 sec and 72ºC 1 min.	146
IDO	Fw` AGA GGA TGC GTG ACT TTG TG 3`<br>Rv` GCAT TTC CAG CCA GAC AGA T 3`	40 cycles 95ºC 0:08 sec, 58ºC 30 sec and 72ºC 1 min.	74

IL-10	Fw´ TTC TTT CAA ACA AAG GAC CAG C 3`<br><br>Rv` CCA AGT AAC CCT TAA AGT CCT GC 3`	40 cycles 95ºC 0:08 sec, 58ºC 30 sec and 72ºC 1 min.	76
TGF-β1	Fw` TGA CGT CAC TGG AGT TGT ACG G 3`<br><br>Rv` GGT TCA TGT CAT GGA TGG TGC 3`	40 cycles 95ºC 0:08 sec, 60ºC 30 sec and 72ºC 1 min.	170
IL-12A	Fw` ATC TCT ATG GTC AGC GTT CCA 3`<br><br>Rv` AGG AGG TAG CGT GAT TGA CAC 3`	40 cycles 95ºC 0:08 sec, 55ºC 30 sec and 72ºC 1 min.	92
EBI-3	Fw` CACTGAAACAGCTCTCGTGGC 3`<br><br>Rv` AAT GAA GGA CGT GGA TCT GGT 3`	40 cycles 95ºC 0:08 sec, 58ºC 30 sec and 72ºC 1 min.	130
β-actin	Fw` ACA CCC GCC ACC AGT TCG 3`<br><br>Rv`CCA CGA TGG AGG GGA ATA CAG 3`	40 cycles 95ºC 0:08 sec, 60ºC 30 sec and 72ºC 1 min.	126

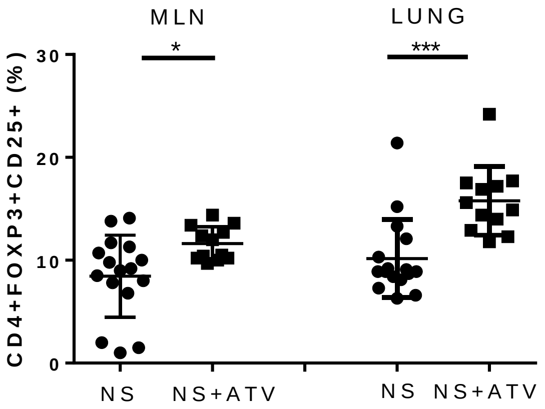

Supporting Information Figure 3. ATV increases the frequency of CD4⁺FOXP3⁺CD25⁺ Tregs in non-sensitized mice. Frequency of $CD4^{+}CD25^{+}FOXP3^{+}$ was evaluated by multi-parametric flow cytometry in MLN and lung from non-sensitized (NS●) and non-sensitized treated with ATV (NS+ATV ■) mice on day 27. Data are shown as mean +/- SD (n = 13 - 17 mice per group pooled from three independent experiments, *p< 0.05, ***p<0.01 Student’s t-test).

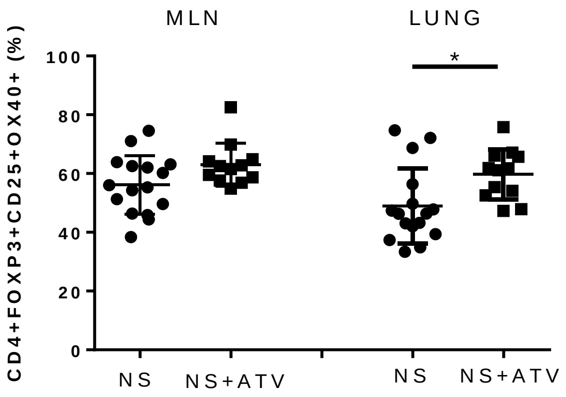

B

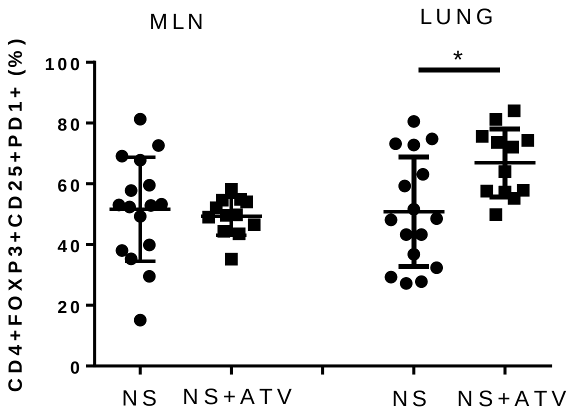

Supporting Information Figure 4. ATV treatment modulates phenotype of Tregs in non-sensitized mice. Frequency of **(A)** $CD4^+FOXP3^+CD25^+OX40^+$ Tregs, **(B)** $CD4^+$ $FOXP3^+CD25^+PD1^+$ Tregs, was measured by flow cytometry in MLN and lung on day 27 in non-sensitized (NS●) and non-sensitized treated with ATV (NS+ATV■) mice. Data are shown as mean +/- SD (n = 13 - 17 mice per group pooled from three independent experiments, *$p < 0.05$ (Student’s t-test).

A

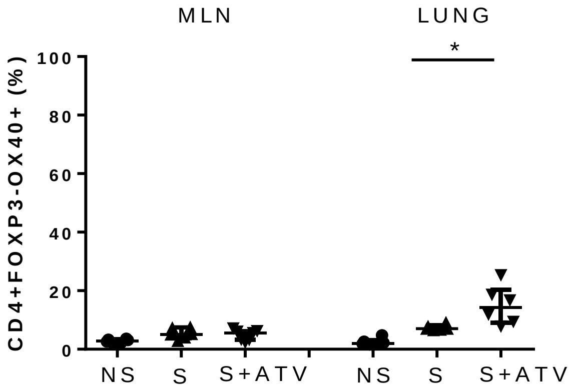

B

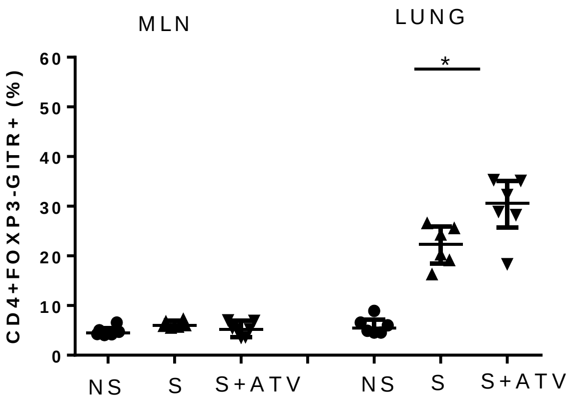

C

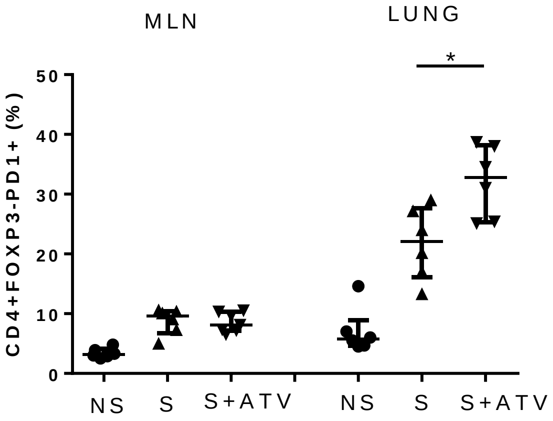

Supporting Information Figure 5. ATV treatment modulates phenotype of Tcon in a murine model of asthma. The frequency of **(A)** $CD4^{+}FOXP3^{-}OX40^{+}$, **(B)** $CD4^{+}FOXP3^{-}GITR^{+}$, and **(C)** $CD4^{+}FOXP3^{-}PD1^{+}$ was evaluated by multi-parametric flow cytometry in MLN and lung from non-sensitized (NS●), sensitized (S▲) and sensitized treated with ATV (S+ATV ▼) mice on day 35. Data are shown as median and interquartile range (n = 6 - 7 mice per group from one experiment. *p<0.05, Mann Whitney test).

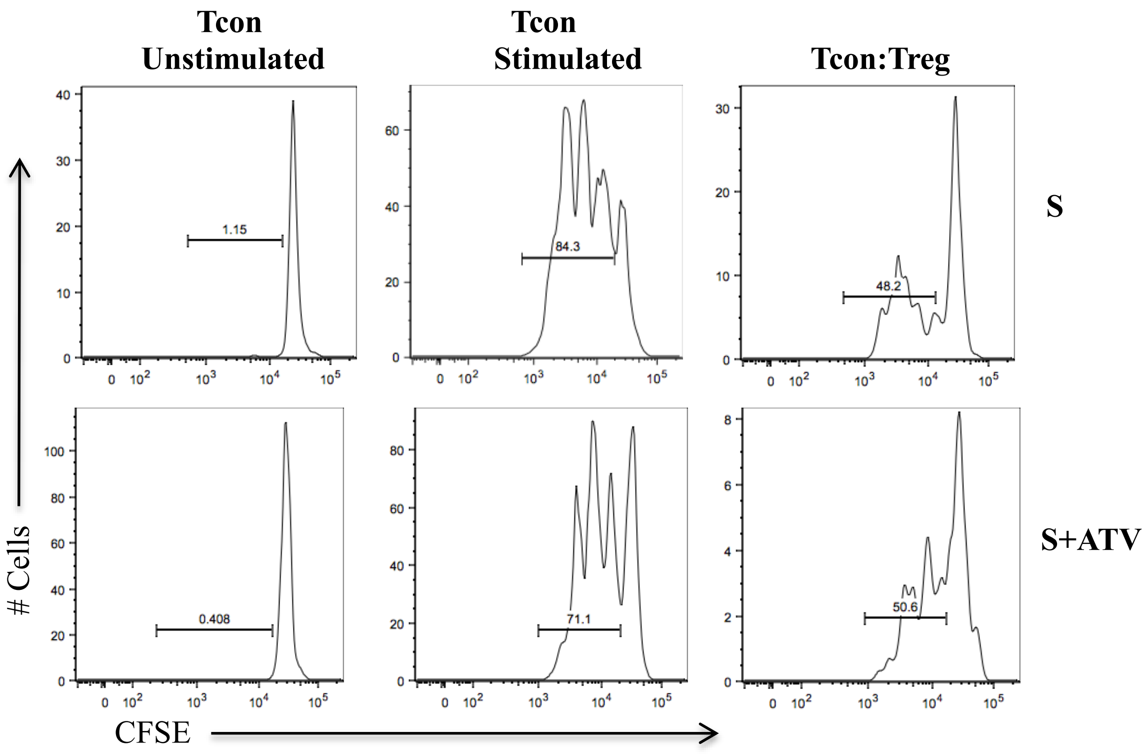

Supporting Information Figure 6. ATV treatment did not increase Treg suppressor capacity in vitro. Representative histogram showing the dilution of CFSE on unstimulated Tcon cells, Tcon stimulated with CD3/CD28/IL-2 or co-cultured with pre-activated Tregs at a ratio of 1:1 and analyzed by flow cytometry after 72 h. The figure represents one experiment from three independent experiments, and the numbers indicate the percentage of division from sensitized mice (S) or sensitized mice treated during 15 days with ATV (S+ATV).

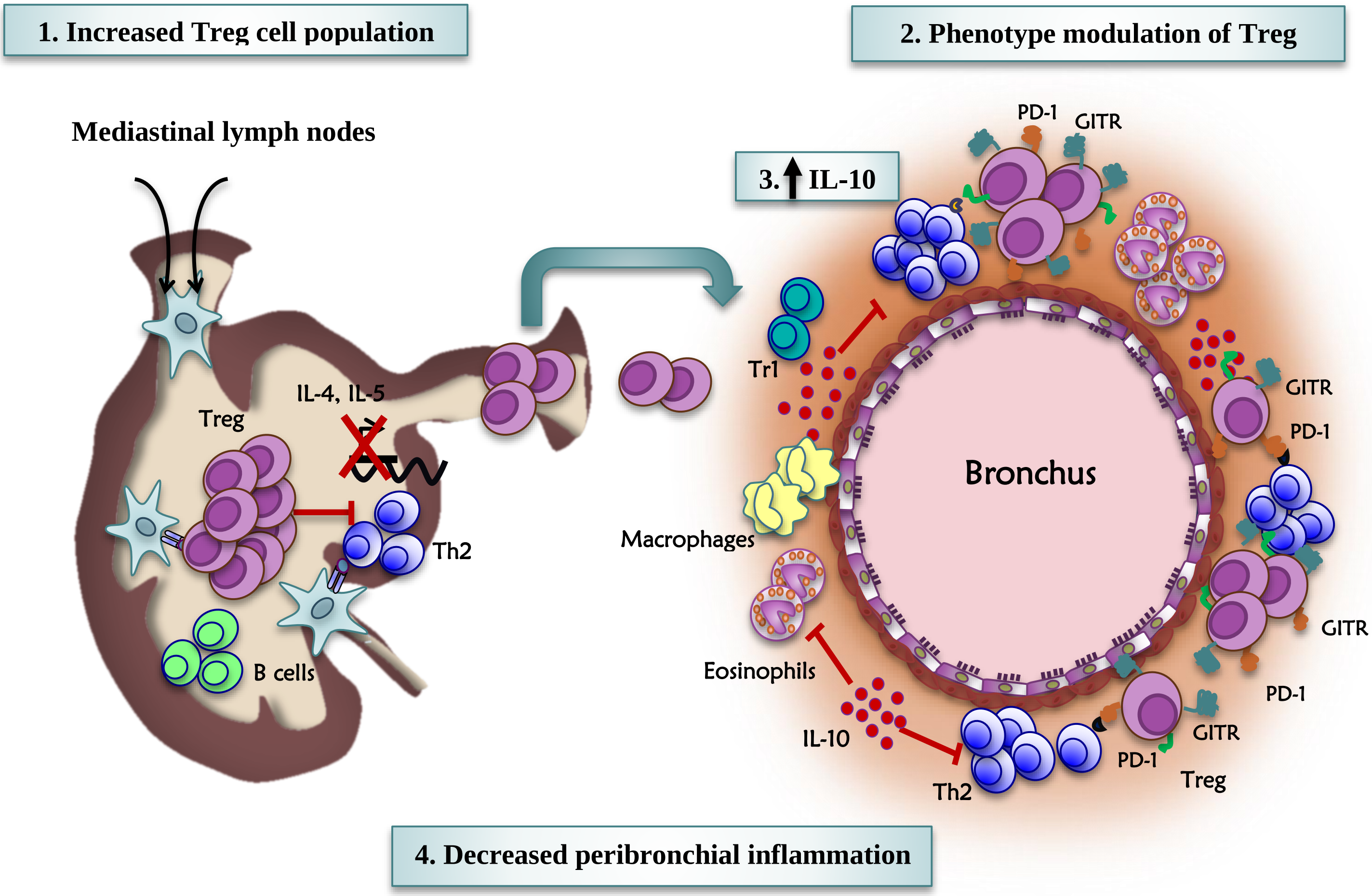

Supporting Information Figure 7. **(1).** The Treg accumulation in the lung-draining lymph nodes could suggest a conversion of T cells into Tregs mediated by ATV; in this tissue, Tregs could be inhibiting the activation of naïve T cells and their differentiation into Th2 cells. **(2).** Higher expression of PD-1 and GITR on both Tcon and Treg indicate the wide effect of ATV on several cellular populations. Modulation of Tregs in the lung suggests that ATV may be enhancing regulatory mechanisms dependent on contact molecules (PD1 and GITR) which mediate tolerance in the respiratory tract. **(3-4)**. Increased levels of IL-10 in the lung, either by Tregs or other regulatory populations (macrophages or Tr1 cells) highlight the immunomodulatory potential of ATV, contributing to the attenuation of the peribronchial inflammation; however further studies are necessary to determine the contribution of Treg cells as part of the immunomodulatory effect of ATV.
